# Numerical study of pedicle screw construction and locking compression plate fixation in posterior pelvic ring injuries: Analyzed by finite element method

**DOI:** 10.1097/MD.0000000000038258

**Published:** 2024-05-17

**Authors:** Jun Zhang, Yan Wei, Jian Wang, Baoqing Yu

**Affiliations:** a Department of Orthopaedics, Pudong New Area People’s Hospital, Shanghai, China; b Department of Surgery, Pudong New Area People’s Hospital, Shanghai, China; c Department of Orthopaedics, Seventh People’s Hospital Affiliated to Shanghai University of Traditional Chinese Medicine, Shanghai, China.

**Keywords:** finite element analysis, locking plate, pedicle screw, pelvic ring injuries

## Abstract

**Background:**

The aim of this study was to compare the biomechanical performance of pedicle screw construction and locking compression plate fixation in posterior pelvic ring injuries analyzed by finite element method.

**Methods:**

A 3-dimensional finite element model of the spine-pelvis-femur complex with ligaments was reconstructed from computed tomography images. An unstable posterior pelvic ring injury was created, which was fixed with a pedicle screw construction or locking compression plate. A follower load of 400 N was applied to the upper surface of the vertebrae to simulate the upper body weight, while the ends of the proximal femurs were fixed. The construct stiffness, the maximum vertical displacement, the maximum posterior displacement, the maximum right displacement, and the overall maximum displacement of the sacrum, and stress distributions of the implants and pelvises were assessed.

**Results:**

The construct stiffness of the pedicle screw model (435.14 N/mm) was 2 times that of the plate model (217.01 N/mm). The maximum vertical displacement, the maximum posterior displacement, the maximum right displacement, and the overall maximum displacement of the sacrum in the pedicle screw model were smaller than those in the plate model (0.919, 1.299, 0.259, and 1.413 mm in the pedicle screw model, and 1.843, 2.300, 1.053, and 2.895 mm in the plate model, respectively). The peak stresses of the implant and pelvis in the pedicle screw model decreased by 80.4% and 25% when compared with the plate model (44.57 and 34.48 MPa in the pedicle screw model, and 227.47 and 45.97 MPa in the plate model, respectively).

**Conclusion:**

The study suggested that the pedicle screw construction could provide better fixation stability than the locking compression plate and serves as the recommended fixation method for the treatment of posterior pelvic ring injuries.

## 1. Introduction

Pelvic ring injuries are severe, with an estimated frequency of 3% of all fractures.^[1]^ Unstable posterior pelvic ring injuries commonly involve disruption of the sacrum, sacroiliac joint, ilium, or a combination of these injuries,^[2]^ mostly leading to vertical and rotational instability of the pelvic ring. These fractures typically suffer from high-energy traumas, such as traffic accidents and falls from heights,^[3]^ and are associated with high rates of morbidity and mortality.^[4]^ Due to destruction of the complex posterior pelvic structures, conservative treatment is usually not an effective therapeutic method. Surgical treatment for unstable pelvic ring injuries has become the gold standard in recent years.^[5]^ However, the therapeutic outcomes of unstable pelvic ring injuries are far from satisfactory, including chronic pelvic pain and restricted activities of daily living.^[6]^ Therefore, it remains a great challenge for orthopedic surgeons to choose an appropriate fixation method for the treatment of unstable pelvic ring injuries.

A great variety of management methods have been investigated for posterior pelvic ring fixation, including external fixation, anterior sacroiliac joint plates, locking compression plates (LCP), transiliac sacral bars, percutaneous sacroiliac screws, and minimally invasive pedicle screw-rod fixators.^[5–8]^ Currently, open-reduction internal fixation is associated with a high rate of wound complications. Close reduction with percutaneous sacroiliac screw fixation is one of the most popular techniques,^[8]^ it has been regarded as a standard method for the definitive treatment of unstable posterior pelvic ring injuries. However, because the complex sacroiliac joint structure involving important blood vessels and nerves, the sacroiliac screw technique requires repeated fluoroscopy with high quality to confirm the appropriate screw position and an experienced surgeon with great surgical skill to avoid neurovascular injuries.^[9]^ Furthermore, many surgeons refuse to expose themselves to X-rays because they are afraid of great radiation exposure. 3D navigation technology has the advantages of reducing radiation exposure during surgery and improving the accuracy of screw implantation.^[10]^ However, navigation equipment is expensive and requires a long learning curve, which makes it difficult to be widely used in various hospitals.

Currently, the primary concern is effective treatment of unstable posterior pelvic ring injuries using minimally invasive technique. LCP may be an effective treatment alternative to stabilize the posterior pelvic ring owing to its advantages of convenience, minimal surgical trauma, and sufficient stability.^[11]^ Despite these advantages, however, LCP has also been associated with a higher incidence of symptomatic hardware,^[12]^ and the difficulty of precontouring the plate according to the irregular structure of the posterior pelvic ring.^[11]^ To avoid these limitations, a minimally invasive pedicle screw-rod fixator has been used for the treatment of posterior pelvic ring injuries.^[13]^ The posterior fixator utilizes 2 pedicle screws connected to a transverse rod for the structural stability of the posterior pelvic ring using a percutaneous invasive technique. Clinical trials have also demonstrated good results with the posterior pedicle screw construct for unstable posterior pelvic ring injuries.^[14]^

Few studies have compared the biomechanical performance of these 2 pelvic fixation techniques. Owing to the variations in bone quality and anatomy, fracture patterns, and fixation location,^[15]^ it is very difficult to compare the biomechanical stability of different implants for the treatment of posterior pelvic ring injuries. In recent years, finite element (FE) analysis has been commonly used to study the advantages and disadvantages of different fixation techniques, which are beneficial for improving clinical results.^[16]^ In the current study, FE analysis was performed to study the biomechanical stability of unstable posterior pelvic ring injuries with the posterior pedicle screw construction and locking compression plate, and to provide a reference for the appropriate selection of fixation devices.

## 2. Methods

This study was approved by the Ethics Committee of the Pudong New Area People’s Hospital.

### 2.1. Finite element modeling

**Figure 1.**
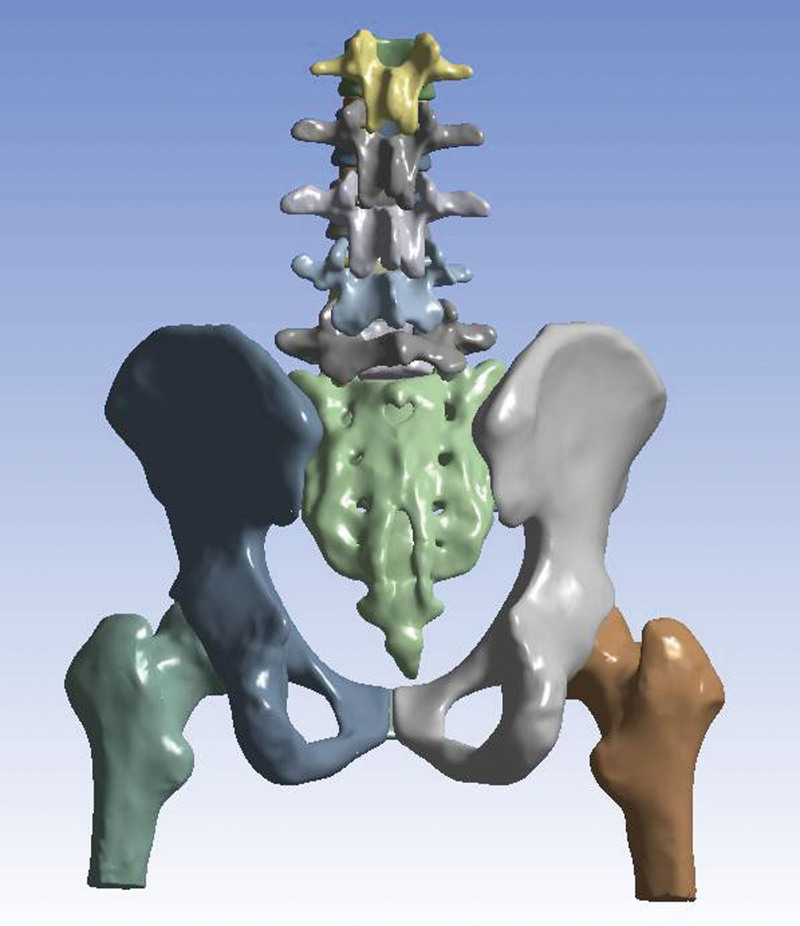
Establishment of geometric model of the spine-pelvis-femur.

A 31-year-old healthy male volunteer (height, 176 cm; weight, 70 kg) with no history of bone diseases, traumas, or deformities was selected and signed an informed consent form for the current study. The spine-pelvis-femur complex, including the L1-L5 lumbar spine, full pelvis, and upper one-third of the femurs was scanned using 128-slice spiral computed tomography (Philips Brilliance, Philips Healthcare, The Netherlands). The slice thickness was set to 1 mm. DICOM data were imported into Mimics software (version 21.0; Materialize, Leuven, Belgium) to reconstruct the geometry of the spine-pelvis-femur based on the segmentation of the computed tomography images (Fig. 1). Smoothing and noise reduction were performed using reverse engineering software Geomagic Studio 2017 (3D System, USA). The normal model was established and modified using the SolidWorks 2017 software (Dassault Systemes, USA). The lumbar spine consisted of the vertebrae (cortical bone, cancellous bone, and posterior elements), and intervertebral discs (annulus fibrosus and nucleus pulposus). The full pelvis consisted of the left ilium, sacrum, right ilium, and pubic symphysis. The bone was segmented into cortical and cancellous bones. The femurs were composed of the upper one-third of the left and right femurs, and the bones were segmented into 2 partitions: the cortical layer and the cancellous core. The spine was connected to the pelvis via articular cartilage in the sacroiliac joint, and the femurs were connected to the pelvis via articular cartilage in the hip joint. To simulate normal condition, thirteen ligaments were constructed: anterior sacroiliac ligament, posterior sacroiliac ligament, interosseous sacroiliac ligament, sacrospinous ligament, sacrotuberous ligament, superior pubic ligament, arcuate pubic ligament, inguinal ligament, iliolumbar ligament, anterior longitudinal ligament, posterior longitudinal ligament, intertransverse ligament, and supraspinous ligament.

**Figure 2.**
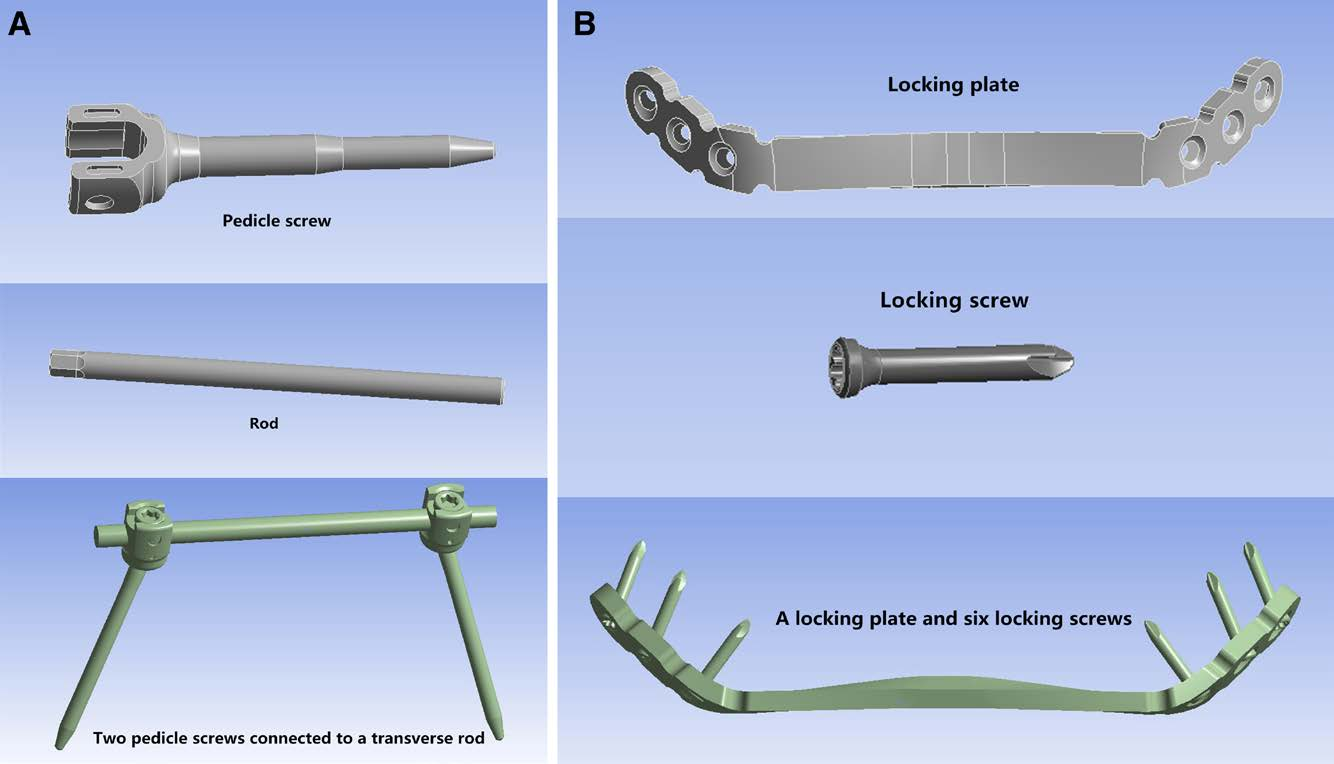
Three-dimensional geometric models of 2 implants. (A) Pedicle screw construction. The 2 pedicle screws had a diameter of 7 mm and a length of 55 mm. The rod was modeled as a 6-mm diameter. (B) Locking compression plate. The locking plate was 4 mm in diameter and 147 mm long. The 6 locking screws had a diameter of 3.5 mm and a length of 20 mm.

Three-dimensional geometric models of the 2 implants (pedicle screw construction and locking compression plate) were reconstructed according to the manufacturer’s specifications using SolidWorks 2017 software. Pedicle screw fixation was constructed using 2 titanium pedicle screws connected to a transverse rod (Fig. 2A). The 2 pedicle screws had a diameter of 7 mm and a length of 55 mm. The rod was modeled as a 6-mm diameter. Plate fixation was performed using a titanium locking plate and 6 locking screws (Fig. 2B). The locking plate was 4 mm in diameter and 147 mm long. The 6 locking screws had a diameter of 3.5 mm and a length of 20 mm.

**Figure 3.**
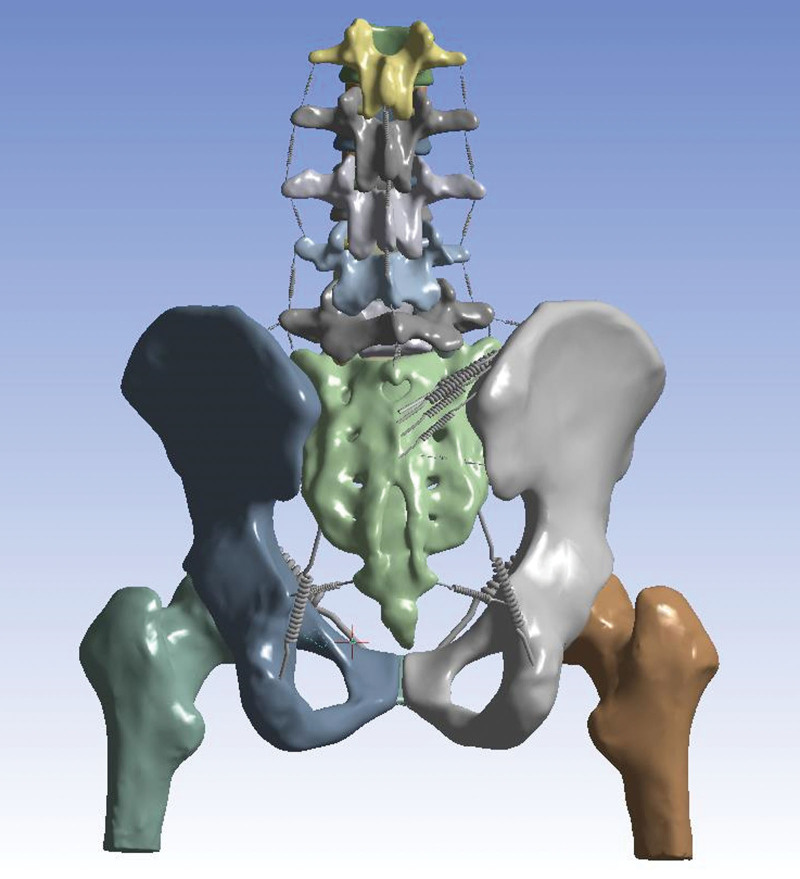
Posterior view of the finite element model of the injured pelvis. After reconstruction of the intact spine-pelvis-femur model, the anterior and posterior sacroiliac ligaments, interosseous sacroiliac ligament, and articular cartilage of the left sacroiliac joint were removed to simulate unstable posterior pelvic ring injuries.

**Figure 4.**
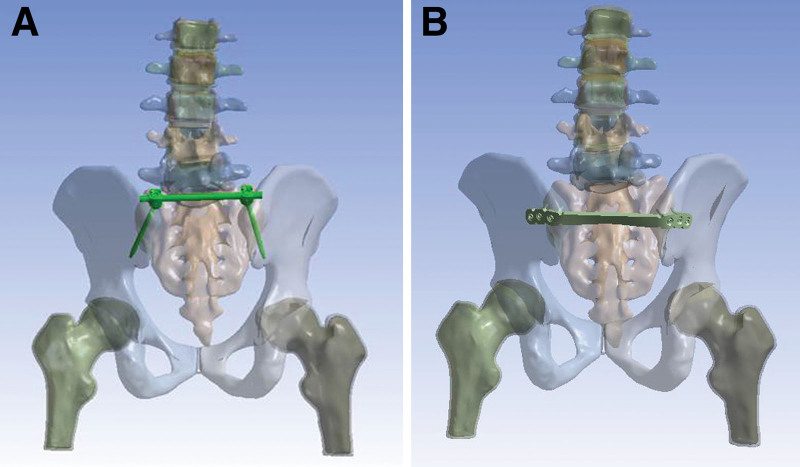
Finite element model of the injured pelvis fixed by 2 implants. (A) Pedicle screw construction. (B) Locking compression plate.

To simulate unstable posterior pelvic ring injuries, the anterior and posterior sacroiliac ligaments, interosseous sacroiliac ligament, and articular cartilage of the left sacroiliac joint were removed (Fig. 3). The implants were positioned according to the clinical experience of an experienced orthopedic surgeon. For pedicle screw fixation, 2 pedicle screws were positioned in the direction from the bilateral posterior superior iliac spine (PSIS) to the anterior inferior iliac spine (AIIS), as shown in Figure 4A. For locking plate fixation, the plate was positioned on the surface of the bilateral PSIS and the 6 screws did not penetrate the sacroiliac joint, as shown in Figure 4B.

The models were imported into the FE software ANSYS Workbench 17 (ANSYS, Inc., USA), through which they were assembled and meshed with 3-dimensional 10-node tetrahedron elements. The number of nodes and elements in the 2 models are listed in Table 1, and the material properties were assigned according to previous reports.^[17]^ To evaluate the accuracy and efficiency of FE models, a mesh convergence test was conducted by adjusting the optimal element size. The maximum von Mises stress on bone was tested and the used convergence criterion was a change of <5%. After the convergence measurement, the mesh size was determined to be 4 mm.

**Table 1:** Number of nodes and elements of the 2 groups of models.

	Pedicle screw construction	Locking compression plate
Node	365285	372010
Element	197740	201452

The FE analysis was conducted using ANSYS Workbench 17 software. All the materials involved in the models were regarded as homogeneous, isotropic, and linear elastic. The ligaments were modeled as tension-only spring elements, and the stiffness coefficients are listed in Table 2. The pedicle screws, rods, locking plates and screws were made of titanium alloy. The material properties of the spine-pelvis-femur complex and implants were obtained from previously published reports^[17–19]^ and are listed in Table 3.

**Table 2:** Material properties of the pelvic and lumbar ligaments.

Ligaments	Stiffness coefficient K (N/mm)
Anterior sacroiliac ligament	700
Posterior sacroiliac ligament	1400
Interosseous sacroiliac ligament	2800
Sacrospinous ligament	1400
Sacrotuberous ligament	1500
Superior pubic ligament	500
Arcuate pubic ligament	500
Inguinal ligament	250
Iliolumbar ligament	1000
Anterior longitudinal ligament	23.75
Posterior longitudinal ligament	26.15
Intertransverse ligament	9.8
Supraspinous ligament	9.8

**Table 3:** Material properties of the spine-pelvis-femur complex and implants.

Materials	Young’s modulus (MPa)	Poisson’s ratio
Lumbar cortical bone	12,000	0.3
Lumbar cancellous bone	100	0.2
Pelvis cortical bone	12,000	0.3
Pelvis cancellous bone	100	0.2
Femur cortical bone	15,000	0.3
Femur cancellous bone	100	0.2
Annulus fibrosus	1	0499
Nucleus pulposus	1	0.499
Posterior elements	3500	0.25
Pubic symphysis	5	0.45
Sacroiliac cartilage	54	0.4
Articular cartilage	100	0.3
Titanium alloy	110,000	0.3

### 2.2. Boundary and loading conditions

With regard to the boundary conditions, the ends of the proximal femurs were fixed in all degrees of freedom to simulate a standing state in a double-leg stance. The interfaces between the following parts were assumed to have frictionless contact, including all of the facet joints, all spinous processes, sacroiliac joints, symphysis pubis, hip joints, the implants and the bones, the pedicle screws and the rod, the locking screws and the locking plate. To validate our FE models, a vertical load of 500 N was applied to the surface of the vertebrae. After the validation of the models, a follower load of 400 N was applied to the upper surface of L1 to simulate the upper body weight.^[17]^

### 2.3. Evaluation criteria

Biomechanical stability was represented by construct stiffness and maximum displacement of the sacrum. Construct stiffness was defined as the ratio of the maximum vertical displacement of the sacrum to the applied load. The *z*-axis represented the upper and lower direction of the spine-pelvis-femur complex, the *y*-axis represented the front and back direction, and the *x*-axis represented the left and right direction. The maximum vertical displacement (*z*-axis), the maximum posterior displacement (*y*-axis), the maximum right displacement (*x*-axis), and the overall maximum displacement of the sacrum were evaluated. In addition, the von Mises stress distributions of the implants and pelvises were analyzed.

## 3. Results

### 3.1. Model validation

**Figure 5.**
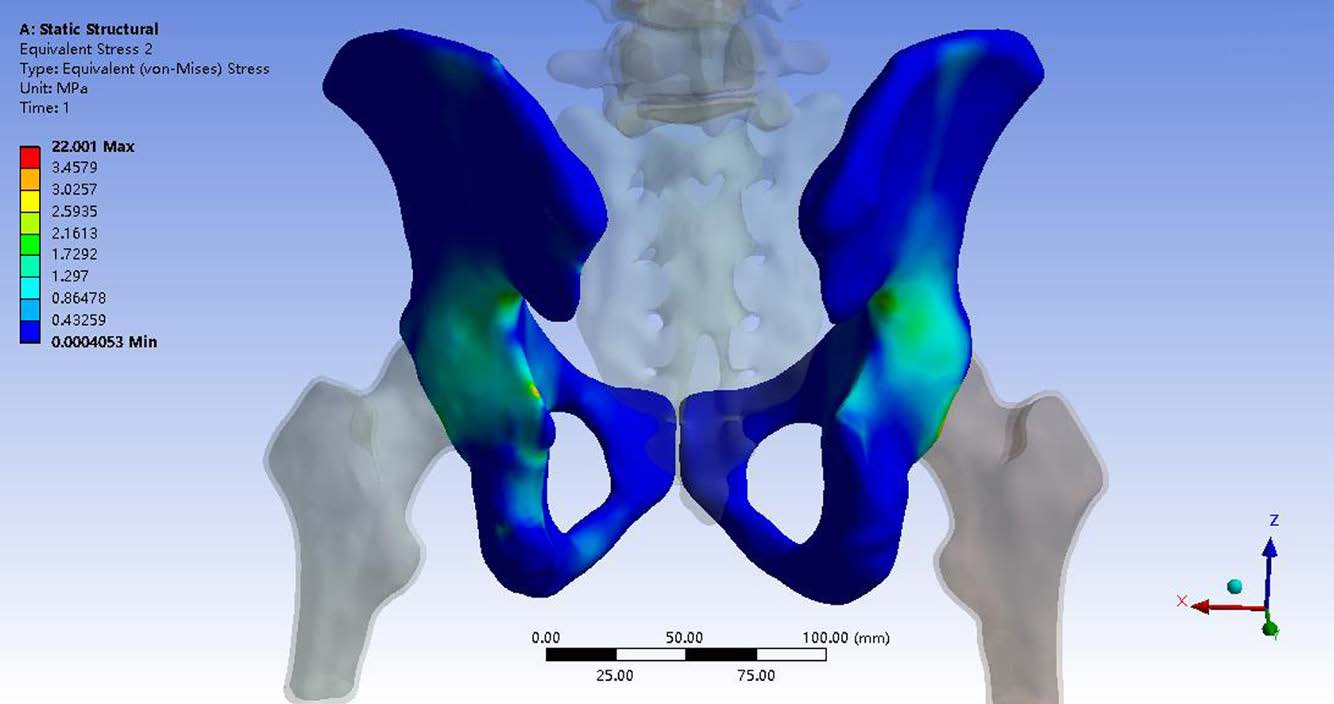
Von Mises stress distribution of the intact pelvis mode under 500 N vertical loading.

For the intact model, the von Mises stress distribution was symmetrical in the sagittal plane, and the stress was concentrated at the greater sciatic notch, and posterior and superior of the acetabulum under 500 N vertical loading. The peak von Mises stress and concentration were in agreement with a previous report,^[17]^ the peak stress in this study was 22.0 MPa (Fig. 5), and the value of the reported data was 13.5 to 25.7 MPa under the same loading. For the injured mode, the construct stiffness of the FE model was 180.03 N/mm under 500 N vertical loading, which was comparable to the cadaveric model (193.2 ± 33.06 N/mm).^[20]^ The results of the intact and injured models demonstrated good agreement with the existing findings, showing that our FE models were satisfactorily validated.

### 3.2. Construct stiffness

Under follower loading of 400 N, the pedicle screw model provided higher construct stiffness (435.14 N/mm) than the plate model (217.01 N/mm). The construct stiffness of the pedicle screw model was 2 times that of the plate model.

### 3.3. Model displacement

**Figure 6.**
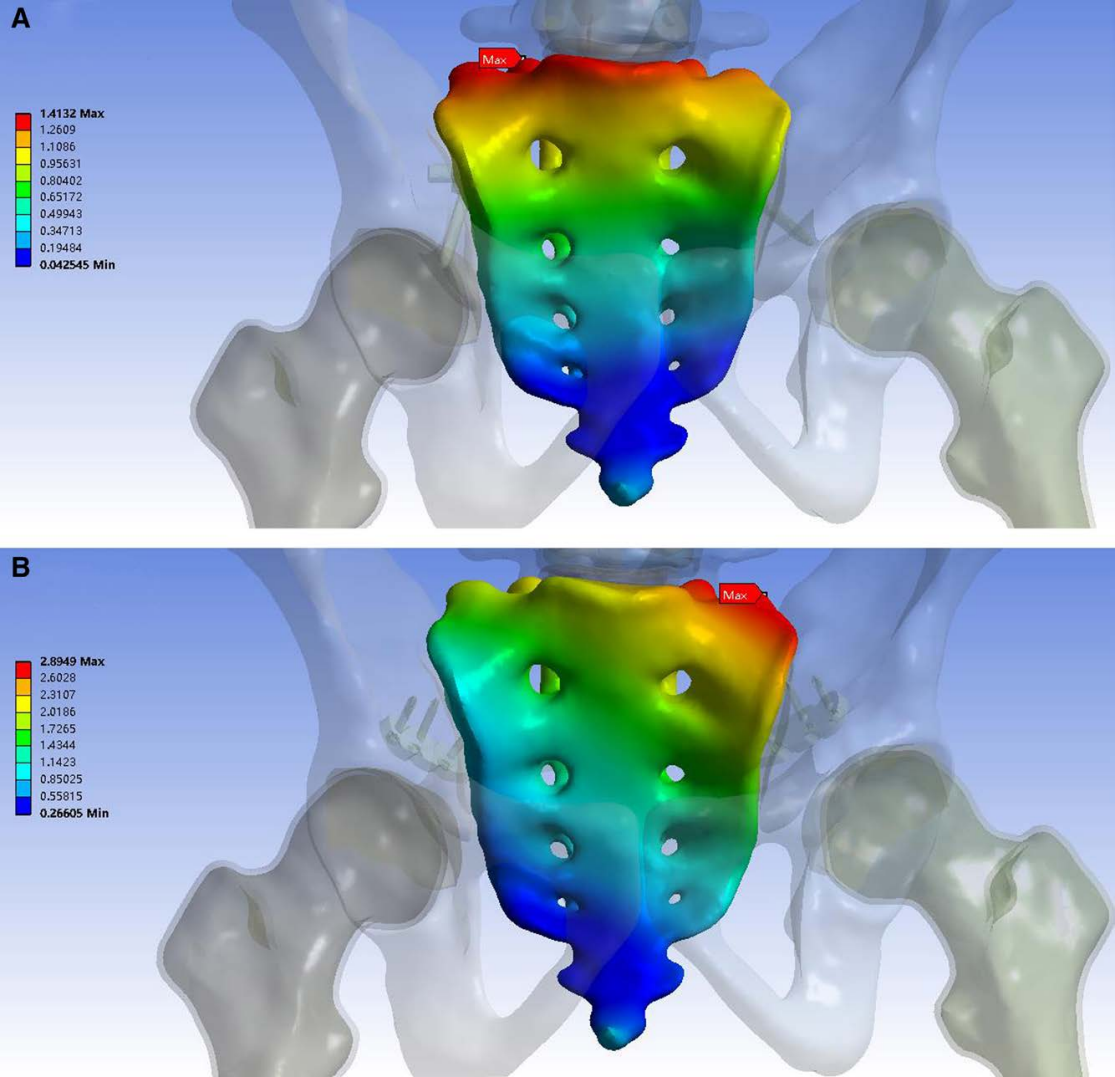
Displacement distribution (mm) of the 2 models under follower loading of 400 N. (A) Pedicle screw construction. The maximum displacement was located at the top of the sacrum on the intact side, and the value was 1.413 mm. (B) Locking compression plate. The maximum displacement appeared at the top of the sacrum on the injured side, and the value was 2.895 mm.

Table 4 and Figure 6 show the displacement distributions of the 2 models under follower loading of 400 N. The maximum displacement of the pedicle screw model was located at the top of the sacrum on the intact side, whereas that of the plate model appeared at the top of the sacrum on the injured side. The pedicle screw model showed a smaller maximum displacement than the plate model. The maximum vertical displacements of the sacrum in the pedicle screw and plate model were 0.919 and 1.843 mm, respectively. The maximum posterior displacements of the sacrum in the pedicle screw and plate model were 1.299 and 2.300 mm, respectively. The maximum right displacements of the sacrum in the pedicle screw and plate model were 0.259 and 1.053 mm, respectively. The overall maximum displacements of the sacrum in the pedicle screw and plate model were 1.413 and 2.895 mm, respectively.

**Table 4:** Parameters results.

Parameters	Pedicle screw construction	Locking compression plate
The maximum vertical displacement of the sacrum (mm)	0.919	1.843
The maximum posterior displacement of the sacrum (mm)	1.299	2.300
The maximum right displacement of the sacrum (mm)	0.259	1.053
The overall maximum displacement of the sacrum (mm)	1.413	2.895
The peak von Mises stress of the implant (MPa)	44.57	227.47
The peak von Mises stress of the pelvis (MPa)	34.48	45.97

### 3.4. Stress distribution

**Figure 7.**
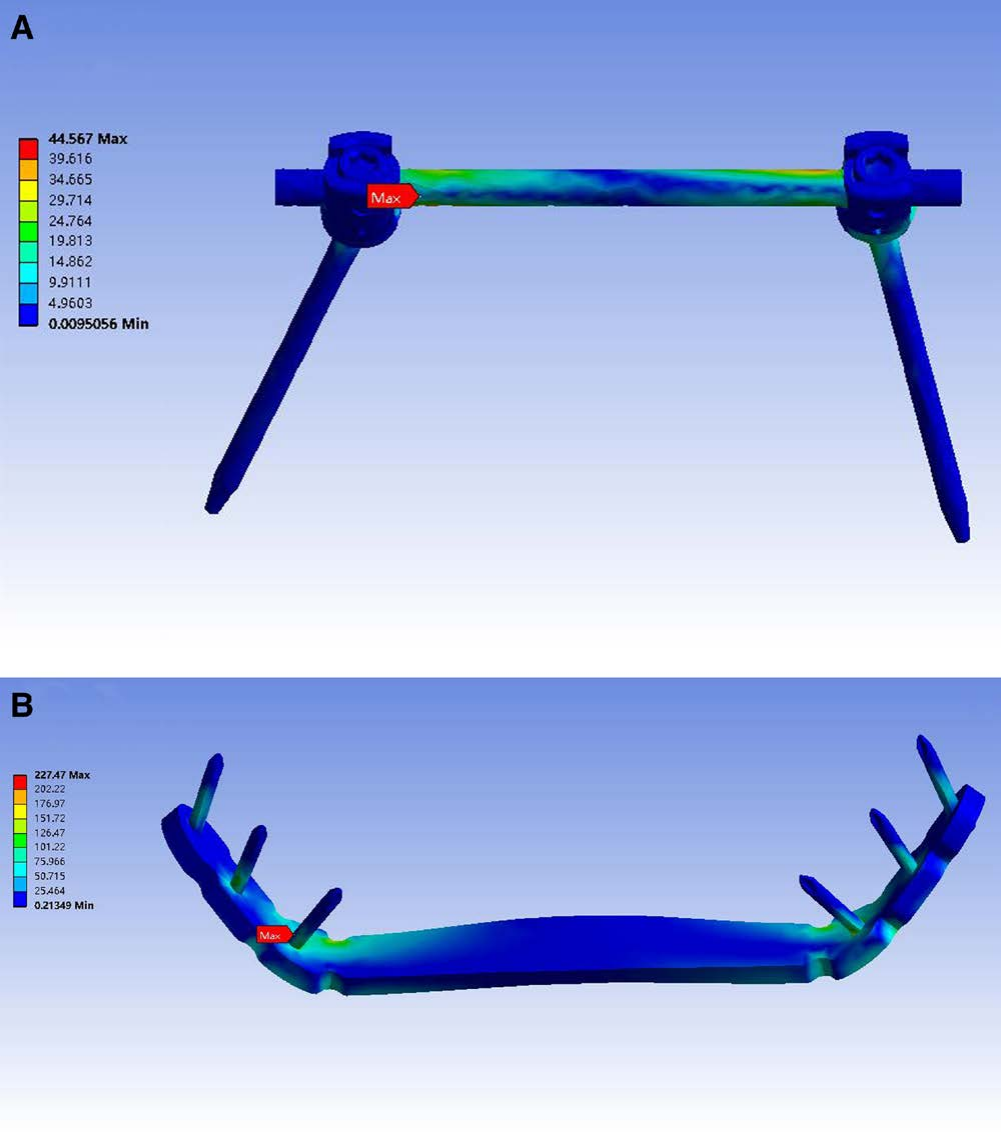
Von Mises stress distribution (MPa) of 2 implants under follower loading of 400 N. (A) Pedicle screw construction. The peak stress appeared at the junction of the pedicle screw and the rod on the injured side, and the value was 44.57 MPa. (B) Locking compression plate. The peak stress was concentrated at the junction of the plate and the locking screw around the fracture site, and the value was 227.47 MPa.

Under follower loading of 400 N, the von Mises stress distributions of the 2 implants are presented in Table 4 and Figure 7. In the pedicle screw model, the peak von Mises stress appeared at the junction of the pedicle screw and the rod on the injured side. In the plate model, the peak stress was concentrated at the junction of the plate and the locking screw around the fracture site. The results showed that the pedicle screw model had a smaller peak von Mises stress than the plate model. The magnitudes of peak von Mises stress in the pedicle screw and plate model were 44.57 and 227.47 MPa, respectively. The peak von Mises stress of the pedicle screw model decreased by 80.4% compared with the plate model.

**Figure 8.**
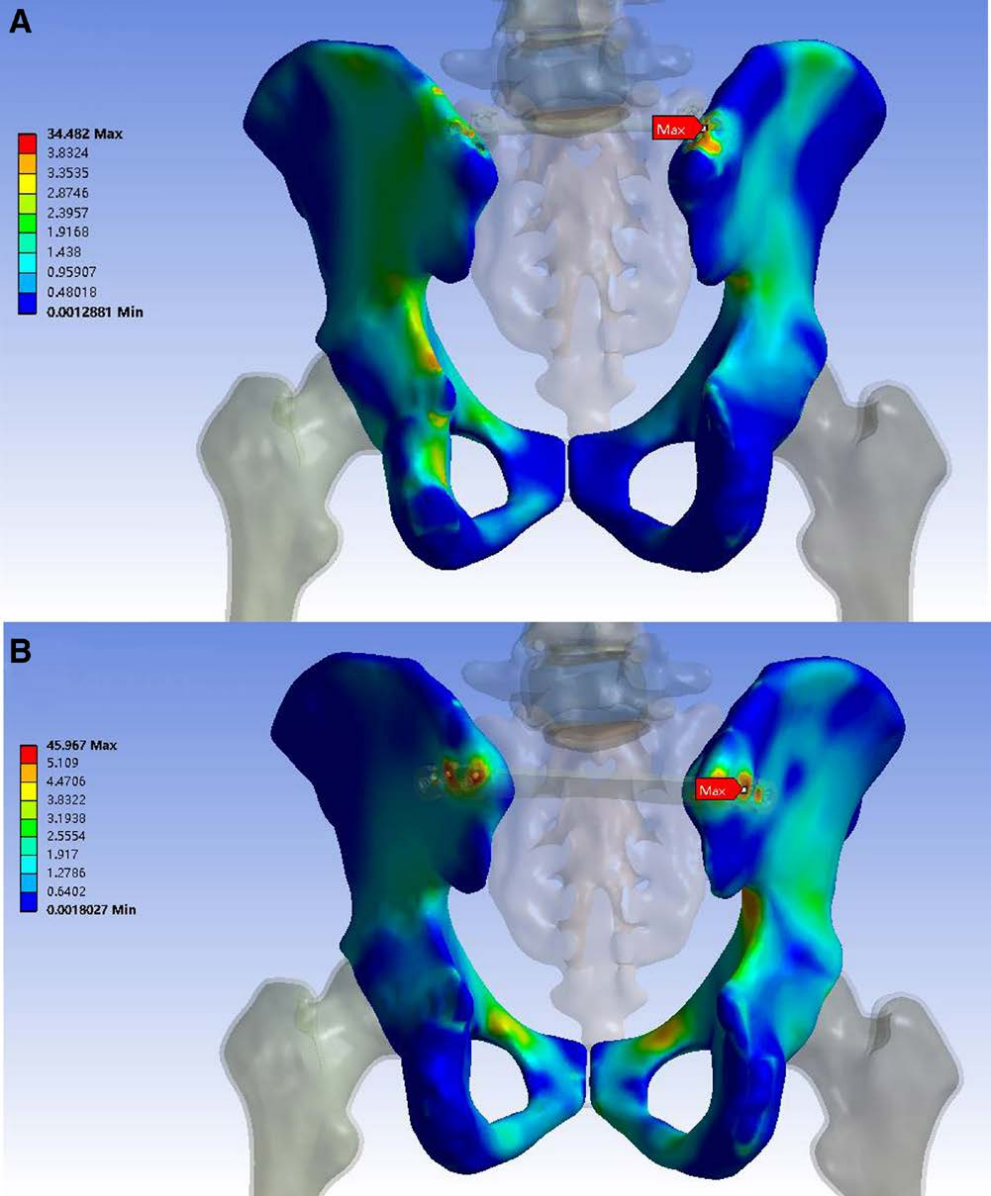
Von Mises stress distribution (MPa) of the pelvis under follower loading of 400 N. (A) Pedicle screw construction. The peak stress was concentrated in the pedicle screw hole on the intact side, and the value was 34.48 MPa. (B) Locking compression plate. The peak stress was concentrated at the middle locking screw hole on the intact side, and the value was 45.97 MPa.

The von Mises stress distribution of the pelvis is presented in Table 4 and Figure 8. In the pedicle screw model, the peak von Mises stress was concentrated in the pedicle screw hole on the intact side. In the plate model, the peak von Mises stress was concentrated at the middle locking screw hole on the intact side. The pedicle screw model had a smaller peak von Mises stress than the plate model, with magnitude of 34.48 and 45.97 MPa, respectively. The peak von Mises stress of the pedicle screw model was 25% less than that of the plate model.

## 4. Discussion

Posterior pelvic ring injuries remain a challenge for orthopedic surgeons, and surgical treatment is necessary to treat these injuries. Various internal fixation methods have been described previously.^[5–8]^ However, each technique has advantages and disadvantages, and no consensus has been reached regarding the optimal method for these injuries. The introduction of percutaneous minimal insertion of sacroiliac screws with limited soft tissue exposure is probably the most common method; however, this technique is technically demanding and limited by potential vascular and neural injuries.^[9,21]^ The LCP technique is an effective treatment of choice for posterior pelvic ring injuries owing to its convenience and minimal trauma. The retrospective data of Hao et al^[11]^ showed that in posterior pelvic ring injuries, LCP fixation could achieve satisfactory radiological and functional results. However, the disadvantages of LCP include limited reduction potential, damage to the threads of screw holes during precontouring of the plate, and potential injury to the nerve and blood vessels when the soft tissue is stripped off.^[6,11]^ To address these limitations, the pedicle screw construction has been introduced as an alternative to treat posterior pelvic ring injuries.^[13]^ Bi et al^[5]^ clinically compared the pedicle screw construction with the LCP in a retrospective study and observed that the size of incision, operation duration and bleeding volume were statistically smaller in the pedicle screw group than in the LCP group.

However, to the best of our knowledge, few biomechanical studies using the FE simulation method have compared the posterior pedicle screw construction with dorsal LCP fixation for the management of unstable posterior pelvic ring injuries. It is difficult to evaluate the biomechanical performance of implants in clinical trials. FE analysis is the preferred method to compare the biomechanics of different implants for the treatment of posterior pelvic ring injuries.^[17,18]^ In the current study, we constructed a 3-dimensional model of sacroiliac joint injury treated with a pedicle screw construction or LCP fixation to evaluate the differences in biomechanical properties. The results of this study found that the 2 pelvic fixation techniques, that is, pedicle screw construction and LCP fixation, significantly affected construct stiffness, model displacement, and stress distribution under the follower load condition. Our results suggest that the construct stiffness of the pedicle screw model was much higher than that of the plate model. Moreover, the maximum displacement in the pedicle screw model was significantly smaller than that in the plate model. In addition, the peak stresses of the implant and pelvis in the pedicle screw model were much lower than those in the plate model.

Evidence suggests that the pedicle screw construction can provide sufficient biomechanical stability in the treatment of unstable posterior pelvic ring injuries. Song et al^[13]^ compared pedicle screw fixation with 2 anterior reconstruction plates in the treatment of Tile C1 pelvic fractures (unilateral sacroiliac joint injury combined with superior and inferior pubic ramus fractures) using FE simulation, and the results showed that the maximum displacements of the ilium and implant in the pedicle screw model were smaller than those in the plate model. Our results showed that the maximum vertical displacement, the maximum posterior displacement, the maximum right displacement, and the overall maximum displacement of the sacrum in the pedicle screw model decreased by 50.1%, 43.5%, 75.4%, and 51.2% compared to the plate model. The smaller maximum displacement of the pelvis represents better fixation stability,^[17]^ and thus, the author concluded that posterior pelvic ring injuries fixed with the pedicle screw construction could achieve sufficient mechanical stability. Dienstknecht et al^[22]^ used freshly frozen human pelvis to simulate an AO type C injury model (unilateral sacroiliac joint disruption combined with pubic symphysis displacement) to compare the pedicle screw construction, 2 sacroiliac screws and 2 ventral compression plates, and observed that differences in the 3-dimensional displacement did not reach statistical significance. The study revealed that the pedicle screw construction had biomechanical stability similar to that of the other 2 internal fixations, and it was considered that the pedicle screw construction might be an alternative to other implants for unstable posterior pelvic ring injuries. In another biomechanical study on pelvic bones,^[23]^ unilateral sacral fractures were simulated and fixed with a modified dual pedicle screw construction or a conventional posterior plate, and the results demonstrated that the mean maximum loads, loads applied to the construct at displacements of 5 and 7.5 mm, and the mean stiffness in the pedicle screw group were significantly higher than those in the posterior plate group, which led the authors to conclude that the use of a modified dual pedicle screw construction was biomechanically stronger than conventional posterior plate fixation in unstable vertical sacral fractures. In our unilateral sacroiliac joint injury model, the construct stiffness of the pedicle screw model was 2 times that of the plate fixation. The construct stiffness of a fixation device is the main determinant of fracture site motion that affects the progression of fracture healing,^[24]^ and clinical studies have demonstrated that a substantial increase in construct stiffness does not seem to interfere with fracture healing.^[14]^ Using the FE model to simulate the Denis II type fracture to compare the pedicle screw construction and 2 sacroiliac screws, Salášek et al^[25]^ proved that the pedicle screw construction could achieve higher stiffness than sacroiliac screws. In detail, the mean stiffness ratio medially and laterally in the pedicle screw model was 75.22% and 57.88%, respectively, while the values in the sacroiliac screw model declined to 46.54% and 44.74%, respectively.

A lower peak stress of fixation devices represents a lower risk of implant failure.^[17]^ In this study, the von Mises stress distributions of the 2 implants were evaluated. The stress in the pedicle screw construction was concentrated at the junction of the pedicle screw and rod, and the stress concentration for plate fixation was found at the junction of the plate and the locking screw. The peak von Mises stress of the implant in the pedicle screw construction decreased by 80.4% compared with plate fixation. From a biomechanical point of view, the pedicle screw model has a lower risk of fatigue failure than the plate model. Nevertheless, it is worth noting that the elastic modulus, yield strength, and ultimate strength of the used titanium alloy were 105 GPa, 921 MPa, and 1024 MPa, respectively,^[26]^ while the peak von Mises stress in the pedicle screw and plate model was 44.57 and 227.47 MPa, respectively. The peak stress of the implant was lower than the yield stress in both models, suggesting that the 2 fixation methods for the treatment of unstable pelvic ring injuries could be safe. The results were in concordance with those of Song et al^[13]^ who conducted a biomechanical study on the treatment of Tile C1 pelvic fractures and found that the maximum stresses of the ilium and implant in the pedicle screw model were less than those in the plate model. In another biomechanical study, Salášek et al^[25]^ demonstrated that the pedicle screw construction experienced lower stress than 2 sacroiliac screws. The von Mises stress ratio of the pedicle screw construction was 139.27%, whereas that of the sacroiliac screws increased to 565.35%.

Recent studies have found that the pedicle screw construction can yield satisfactory clinical results in treating unstable posterior pelvic ring injuries. In a retrospective study, Hua et al^[14]^ reported that in unstable pelvic ring injuries, 23 patients were treated with a minimally invasive anterior internal pelvic fixator with or without a posterior pedicle screw construction and found an excellent or good rate of 82.6% in postoperative radiographic outcomes according to the Matta criteria and 87% in clinical results at 6 months postoperatively according to the Majeed scores. The results were in concordance with those of Wu et al^[27]^ who conducted a retrospective analysis of 23 unstable pelvic ring injuries using anterior internal pelvic fixator with or without a posterior pedicle screw construction and observed that an excellent or good rate of 87% was acquired according to the radiological Matta criteria and 91.3% in clinical results at 12 months postoperatively according to the Majeed scores. Similar retrospective data by Wang et al^[28]^ found that an excellent and good rate of 89.7% in both radiological Matta criteria and clinical Majeed scores in a series of 29 posterior pelvic ring instabilities with the pedicle screw construction. The results were compatible with the data of Bi et al^[5]^ who performed a retrospective analysis of unstable posterior pelvic ring fractures and found an excellent rate of 83.3% in clinical Majeed scores when a pedicle screw-rod fixator was used. In a prospective study, Salášek et al^[29]^ reported that in a series of 64 Tile C1 pelvic fractures, no significant difference in the clinical Majeed scores was found between the pedicle screw construction group and the 2 sacroiliac screws groups. However, 2 patients developed iatrogenic neurological injuries in the sacroiliac screws, while no intraoperative complications were associated with the pedicle screw construction.

The transiliacal internal fixator, which was initially described by Füchtmeier et al^[30]^ in a prospective study that included 31 patients with vertical shear injuries of the pelvis, using two 7.0 mm pedicle screws connected to a transverse rod, was proven to be a minimally invasive technique with a very low rate of neurovascular injuries for stabilization of sacroiliac joint ruptures and sacral fractures. In a study by Füchtmeier et al, pedicle screws were placed into the PSIS in the cranio-caudal direction and parallel to the superior gluteal line. To obtain sufficient biomechanical stability by internal fixation with a pedicle screw-rod system, a pedicle screw with a greater diameter is recommended.^[30]^ In a radiographic morphometric study, Schildhauer et al^[31]^ found that the supraacetabular bone canal from the PSIS to the AIIS to place pedicle screws demonstrated the optimal path for lumbopelvic fixation in the treatment of spinal and pelvic ring injuries. The bone corridor allowed placement of the implant with a length of 141 mm in male and 129 mm in female patients, and screws with 8 mm diameter in male and 6 to 7 mm in female patients. Schmitz et al^[32]^ confirmed the results of Schildhauer and proved that Schanz screws placed into the supraacetabular bone canal could reach a length of up to 135 mm (mean 100 ± 20 mm), which led the authors to conclude that placement of the Schanz screws from the PSIS to the AIIS could provide greater mechanical stability for fragility fractures of the pelvis with vertical and rotational instability. In our study, two 7.0 mm pedicle screws of 55 mm length were inserted in the direction from the PSIS to the AIIS.

However, this study has some limitations. First, the material properties of the bones and implants were simplified as homogeneous, isotropic, and linearly elastic. Second, the muscles were not included in the models, which could not fully reflect real conditions. Finally, only static loads were applied to the spine-pelvis-femur complex for the FE analysis. In reality, the spine, pelvis, and femur, are exposed to complex forces and moments during normal activity. Despite this, the FE model used in this study was comparable to that used in previous in vitro studies.

## 5. Conclusion

The current study compared the biomechanical properties of pedicle screw construction and LCP fixation in unstable posterior pelvic ring injuries. According to the FE analysis, the use of pedicle screw fixation could increase construct stiffness, decrease displacement, and reduce stress in the implant and pelvis. Therefore, the pedicle screw construction could provide better fixation stability than LCP fixation and serves as the recommended fixation method for the treatment of posterior pelvic ring injuries.

## Author contributions

**Conceptualization:** Jun Zhang, Baoqing Yu.

**Data curation:** Yan Wei.

**Formal analysis:** Jun Zhang, Yan Wei.

**Funding acquisition:** Jun Zhang.

**Methodology:** Jun Zhang, Jian Wang.

**Software:** Jun Zhang, Yan Wei, Jian Wang.

**Writing—original draft:** Jun Zhang.

**Writing—review & editing:** Jun Zhang, Yan Wei, Baoqing Yu.
